# Energy Efficient Policies for Data Transmission in Disruption Tolerant Heterogeneous IoT Networks

**DOI:** 10.3390/s18092891

**Published:** 2018-08-31

**Authors:** George Stamatakis, Elias Z. Tragos, Apostolos Traganitis

**Affiliations:** 1 Institute of Computer Science, Foundation for Research and Technology Hellas, N. Plastira 100, 70013 Heraklion, Greece; elias.tragos@insight-centre.org (E.Z.T.); tragani@ics.forth.gr (A.T.); 2 Insight Centre for Data Analytics, University College Dublin, Belfield, D04 V1W8 Dublin 4, Ireland

**Keywords:** Internet-of-things, heterogeneous networks, data transmission, disruption tolerant IoT, dynamic programming

## Abstract

The Internet-of-things facilitates the development of many groundbreaking applications. A large number of these applications involve mobile end nodes and a sparsely deployed network of base stations that operate as gateways to the Internet. Most of the mobile nodes, at least within city areas, are connected through low power wide area networking technologies (LPWAN) using public frequencies. Mobility and sparse network coverage result in long delays and intermittent connectivity for the end nodes. Disruption Tolerant Networks and utilization of heterogeneous wireless interfaces have emerged as key technologies to tackle the problem at hand. The first technology renders communication resilient to intermittent connectivity by storing and carrying data while the later increases the communication opportunities of the end nodes and at the same time reduces energy consumption whenever short-range communication is possible. However, one has to consider that end nodes are typically both memory and energy constrained devices which makes finding an energy efficient data transmission policy for heterogeneous disruption tolerant networks imperative. In this work we utilize information related to the spatial availability of network resources and localization information to formulate the problem at hand as a dynamic programming problem. Next, we utilize the framework of Markov Decision Processes to derive approximately optimal and suboptimal data transmission policies. We also prove that we can achieve improved packet transmission policies and reduce energy consumption, extending battery lifetime. This is achieved by knowing the spatial availability of heterogeneous network resources combined with the mobile node’s location information. Numerical resultsshow significant gains achieved by utilizing the derived approximately optimal and suboptimal policies.

## 1. Introduction

The Internet-of-things (IoT), i.e., the network of tangible objects with embedded sensors, actuators and network interfaces has had a profound impact on key sectors of economy [1]. Through an ever growing set of applications that target the diverse needs of both urban and rural environments vast amounts of data are collected and analyzed so that production and service processes are streamlined through informed decisions and controls. Examples of such IoT applications include, but are not limited to, smart cities, smart factories, smart agriculture, parking and traffic management, water management, e-Health, environment monitoring and education [2].

In a large number of the aforementioned IoT applications utilization of *mobile* sensor nodes is preferable or unavoidable [3]. This is especially true for applications that involve wildlife and environmental monitoring within large, probably uncharted, areas where the deployment of base stations, that operate as gateways to the Internet, is typically sparse. It is also true for applications that utilize mobile devices for data collection such as smart phones and smart vehicles. However, mobility and sparse deployment of base stations comes at the cost of intermittent network connectivity and large delays. Studies and experiments have also shown that interference in rural environments can also cause severe impact on the connectivity and the performance of the networks [4,5]. Delay Tolerant Networks (DTNs) is a class of network architectures that could address the lack of continuous network connectivity in IoT networks [6]. On the other hand, DTNs follow a store, carry and forward pattern for data routing and turn the limited buffer space of mobile sensors to a valuable resource. Furthermore, a long battery lifetime is a prerequisite for most IoT applications due to the difficulties associated with charging or replacing batteries once the devices have been deployed. This turns energy consumption into another limited and thus valuable resource. To tackle both the problem of energy efficiency and intermittent connectivity, it has been considered [7] to equip mobile IoT devices with network interfaces that are heterogeneous. Wireless interfaces with a long communication range increase network coverage while short-range interfaces may reduce the amount of energy consumed by radio transmissions which are a major source of energy consumption [8].

In this work we deal with the problem of finding an energy efficient packet transmission policy for a mobile node that participates in a heterogeneous disruption tolerant IoT network. Typically, whenever a mobile node comes within the communication range of one or more base stations it transmits all the packets in its backlog using the most energy efficient wireless interface available at that time. However, this packet transmission policy completely disregards the potential availability of more energy efficient wireless networks in nearby locations and thus does not capitalize on the node’s mobility in order to reduce the total energy consumed in packet transmissions over a long period of time. This some times results to the well-known “ping-pong effect” when the mobile node movement is not considered in the handover decisions [9]. In this work we consider the advantage offered by a map with spatial information regarding network coverage within the area traversed by the mobile node in devising an energy-efficient packet transmission policy. In real-world environments, there is temporal variation of the wireless network availability, which might induce inaccuracy in the network availability map that is a central point of the proposed system. However, even in this case, the proposed scheme does not become inapplicable, since temporal changes can be dealt with other network mechanisms or the network availability map can be constructed using longer term average availability measurements. To the best of our knowledge we are the first to formulate the problem at hand as a dynamic programming problem and utilize the framework of Markov Decision Processes (MDP) to derive approximately optimal and suboptimal packet transmission policies. Furthermore, we exhibit that knowledge of the spatial availability of heterogeneous network resources combined with mobile node’s location information leads to improved packet transmission policies that significantly reduce energy consumption and thus extend battery lifetime.

The remainder of this paper is organized as follows. In Section 2 we present past work related to the problem at hand. In Section 3 we present the specifics of the problem, our model and it’s key assumptions. Section 4 presents a formulation of the problem as a MDP and iterative algorithms for the derivation of optimal and suboptimal policies. Section 5 presents numerical results for the evaluation of the proposed policies. Finally, we conclude with Section 6.

## 2. Related Work

Although it has been clear for many years that a major challenge for the IoT is its ability to cope with the high network dynamism, it is only recently that DTNs have been identified as potential enablers for the IoT. For example in [10] the authors develop a Bundle Protocol (BP) convergence layer for IEEE 802.15.4-based networks and evaluate it through a use case that involves an elevator carrying a sensor. Another example is [11] where a basic implementation of a BP binding for Constrained Application Protocol (CoAP) is proposed, as a means to enable delay tolerant IoT, and some first experimentation results are presented that validate the feasibility of the approach.

There are numerous other works where IoT, Wireless Sensor Networks and DTNs coexist. In [12] the authors present opportunistic IoT, which addresses information dissemination and sharing within and among opportunistic communities which are formed by the movement and opportunistic contact among humans bearing smart devices. In [13] the authors discuss the notion of a Challenged IoT and propose Direct Interaction with Smart Challenged Objects, a technique that enables objects to convey their interface directly to other mobile users. In [14] the authors present a novel buffer management algorithm for DTNs, called SmartGap, that estimates the value of each packet by means of its contribution to the reconstruction of the original signal to be transmitted. More recently, in [15] the authors present a collection of survey papers related to DTN routing as well as most of the aforementioned works that utilize DTNs in order to tackle IoT related problems.

A work that is more closely related to ours, in terms of the network architecture considered, is [7] where the authors consider a DTN with intermittent connection between mobile nodes that are equipped with two radio interfaces. The first one is a long-distance radio which is used for neighbor discovery while the second one is a 802.11 interface used for data transmission. Furthermore, the authors consider using battery powered stationary nodes, called “Throwboxes” in order to increase the number and frequency of contact opportunities for the mobile nodes. The authors tackle the problem of reducing the energy consumed in neighbor discovery and, more specifically, their objective is to maximize the number of packets being forwarded by Throwboxes under an average power constraint by utilizing mobility prediction. Similar to [7] the authors in [16] consider solar powered mobile nodes in a DTN that again have two wireless interfaces. The first one is a 802.15.4 interface which is used by a power management module that is responsible for negotiating complex wakeup rules whenever a potential contact with another mobile node is feasible, while the second one is a 802.11a used for data transmission. The focus of this study is again to minimize energy consumption related to the discovery of communication opportunities. Unlike the previous two works we utilize both the long and short range interface for data transmission while we do not consider the problem of neighbor discovery.

Finally, in [17] the authors provide a comprehensive survey of DTN related works that consider the use of throwboxes as performance enhancers. The works surveyed [17] consider a wide range of problems related to the use of throwboxes such as their cardinality, placement criteria, buffer management, network topology, mobility characteristics of nodes, application requirements, and cooperation of throwboxes among themselves and with other nodes. However, none of these works works addresses the same problem or considers a system model that is similar to the one we consider.

To our knowledge our work is the first one to address the problem of finding an energy efficient packet transmission policy for a mobile node that participates in a DTN IoT network where mobile nodes are equipped with two wireless interfaces for data transmission.

## 3. System Model and Definitions

**Figure 1:**
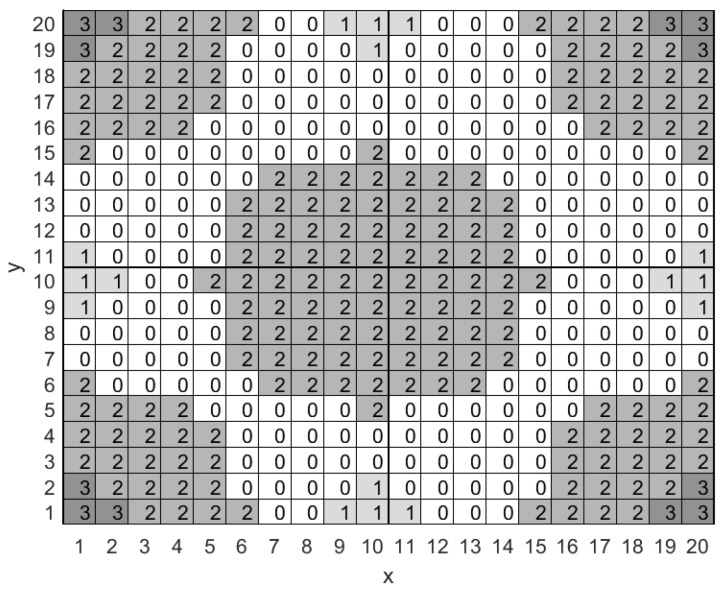
Example of the network availability map for a rectangular surveillance area divided in square-shaped cells.

We consider a rectangular surveillance area that is subdivided into smaller square-shaped cells as shown in Figure 1. Each cell within the surveillance area is identified by the Cartesian coordinates of its center, i.e., by a pair (x,y), where x=1,2,⋯,X and y=1,2,⋯,Y. Furthermore, each cell *may* host a Low Power Wide Area Network (LP-WAN) base station [18], featuring wide-area connectivity for relatively low power and low data rate devices that is not provided by legacy wireless technologies. LP-WAN base stations may use technologies such as LoRa [19,20], SigFox [21] or NB-IoT [22]. What is more, each cell may also host a Low Power Personal Area Network (LP-PAN) base station, i.e., a base station that supports higher bit rates and lower power consumption albeit at a much sorter range compared to an LP-WAN base station. An example of a LP-PAN base station could be an 802.15.4 base station [23]. Finally, cells may host both types of base stations. Let $A^{p}$ and $A^{w}$, be integer matrices whose elements take values in the set {0,1} to indicate that a specific cell lies within the communication range of at least one LP-PAN and LP-WAN base station, respectively, e.g., $a_{xy}^{w}=1$ ($a_{xy}^{w}=0$) indicates that cell (x,y) lies (doesn’t lie) within the communication range of one or more LP-WAN base stations. We note here that the optimal way to choose the base station to associate with, among many base stations of the same type, is beyond the scope of this work. Given $A^{p}$ and $A^{w}$, we may compute a network availability matrix as $A^{n}=A^{p}+2A^{w}$ which enables us to present network availability in a single figure (see Figure 1). More specifically, cells that lie exclusively within the communication range of at least one LP-PAN or at least one LP-WAN base station are respectively marked with 1 and 2, while cells that lie within the communication range of both types of base station are marked with a 3. Cells that lie outside the communication range of all base stations lack network connectivity and are marked with a 0.

We note here that in this work we do not consider the temporal variation of the network availability map, however, this does not necessarily render our proposed scheme inapplicable. The key idea here is that the efficiency, and not the applicability, of the derived packet transmission policy depends on the accuracy of the network availability map, which in turn depends on the process and specifications of its construction. For example, to mark a specific network type as available in a specific cell of the network availability map one could pose the most stringent constraint on its temporal availability, thus producing a map that rarely changes as time passes by. What is more, temporal changes in network availability can be dealt with other network mechanisms, such as packet retransmissions, although we do not consider such mechanisms in this work. We do expect though that, even in the absence of such mechanisms, the proposed packet transmission policy will not result in a complete breakdown of network service although it will definitely be suboptimal. We emphasize here that utilization of mobile nodes that may offer opportunistic connectivity, as well as the creation of the network availability map is beyond the scope of our work. For recent work, related to the subject of network availability map creation, such as network topology inference and spatial availability and reliability of wireless links the interested reader may refer to [24,25,26,27].

Next, we consider a mobile node that is appropriately equipped with a LP-PAN interface, a LP-WAN interface and sensors. The mobile node performs a two-dimensional random walk within the surveillance area collecting data either through its sensory equipment or by reception of packets from its neighboring nodes. We assume that the random walk evolves in stages of equal duration indexed by k=0,1,⋯. At the beginning of the *k*-th stage the mobile node identifies its current cell $\left(x_{k},y_{k}\right)$ through some localization mechanism that we assume to be exact and the number of packets $m_{k}$ in its backlog. We assume that the node’s backlog is finite with a maximum capacity of *M* packets. Values $x_{k}$, $y_{k}$ and $m_{k}$ constitute the state of the system at stage *k*, which we denote with the column vector $s_{k}={\left[x_{k},y_{k},m_{k}\right]}^{T}$. The state of the system summarizes all past information that is relevant for future optimization.

Given its current state the mobile node must make a decision regarding the wireless interface to use and the number of packets to transmit or drop. We denote the set of all possible decisions, termed controls, with $U=\{\left(u^{w},u^{p},u^{m}\right):u^{w}\in \left\{0,1\right\},\;u^{p}\in \left\{0,1\right\},\;u^{w}+u^{p}\neq 2,\;u^{m}=1,2,\cdots ,M\}$, where $u^{w}$, $u^{p}$ are binary variables indicating the selection of the LP-WAN and LP-PAN interface respectively and $u^{m}$ is the number of packets to transmit or drop from the backlog. Equation $u^{w}+u^{p}\neq 2$ indicates that the sensor is not allowed to use both interfaces at the same stage by prohibiting both $u^{w}$ and $u^{p}$ to be equal to one at the same stage. On the other hand, if both $u^{w}$ and $u^{p}$ are zero we distinguish two cases based on whether $u_{m}$ is zero or a positive integer. In the first case the mobile node will not transmit any packets while in the latter it will drop $u_{m}$ packets.

For each system state only a subset of the decisions in *U* will be available to the mobile node, unless of course both types of network are available in the current cell and the backlog is full. To determine the set of available controls in each state we note that given the current state of the system $s_{k}$, and the network availability map, the mobile node can determine the available types of network in the current cell and restrict the range of values for $u^{w}$ and $u^{p}$ accordingly. Furthermore, the number of packets in the backlog $m_{k}$ is an upper bound for the number of packets the mobile node can transmit or drop, i.e., $u^{m}\leq m_{k}$. Additionally, we assume that the mobile node preemptively transmits or drops packets so that the backlog can always accommodate the maximum number of new packets $W_{m}^{\max}$ that may be generated during the current stage. This queue management strategy, although atypical, results in a significant state space reduction since dropped packets are not explicitly accounted for in the state vector. To enforce the latter constraint on the set of controls we require that $u^{m}\geq \max\left\{0,\;m_{k}+W_{m}^{\max}-M\right\}$. Finally, to simplify notation, we note that the set of available controls in any state $s_{k}$ remains unchanged across stages, i.e., it is independent of index *k*. Thus we may drop index *k* from vector $s_{k}$ and its elements and summarize the constraints discussed above for any state $s={\left[x,y,m\right]}^{T}\in S$ as follows,

(1) $$\begin{array}{cc} U\left(s\right)=\{\left(u^{w},u^{p},u^{m}\right):u^{w}\in \left\{0\right\}\cup \left\{a_{x,y}^{w}\right\},u^{p}\in & \left\{0\right\}\cup \left\{a_{x,y}^{p}\right\},\\ & \;\max\left\{0,\;m+W_{m}^{\max}-M\right\}\leq u^{m}\leq m,\;u^{m}\in N\}, \end{array}$$

where $u^{m}$ represents packet drops only when no network is available, i.e., when both $u^{w}$ and $u^{p}$ are equal to zero and $u^{m}>0$.

At the beginning of the next stage the state of the mobile node will change as a result of the control $u_{k}=\left(u_{k}^{w},u_{k}^{p},u_{k}^{m}\right)\in U\left(s_{k}\right)$ applied at the *k*-th stage and the random variables of the system. A random variable of the system is $W_{k}^{m}$, which is an integer random variable indicating the number of new packets added in the backlog during the *k*-th stage by the sensory equipment of the mobile node. We also consider the pair of integer random variables $W_{k}^{\Delta x}$ and $W_{k}^{\Delta y}$ that represent the random displacement of the mobile node along the *x* and *y* axis respectively. We assume that the system’s random parameters are independent of their values in previous stages. Furthermore, we assume that $W_{k}^{m}$ is uniformly distributed between 0 and $W_{m}^{\max}$ for all cells within the surveillance area. On the other hand, the probability distributions of $W_{k}^{\Delta x}$ and $W_{k}^{\Delta y}$ are not the same for all $\left(x_{k},y_{k}\right)$. This dependence on $\left(x_{k},y_{k}\right)$ stems from the fact that the mobile node will follow a different moving pattern in case it reaches a cell that lies on the border of the surveillance area. More specifically, the node will either remain in the same cell or move to a neighboring one as long as this transition does not cross the boundaries of the surveillance area. However, if the mobile node is in a cell that lies on the boundary of the surveillance area it will either remain on the boundary or bounce back into the surveillance area. Thus, for all k=0,1,⋯, we have that $W_{k}^{\Delta x}$ is uniformly distributed in the set {−1,0,1} given $1<x_{k}<X$, uniformly distributed in the set {0,1} given $x_{k}=1$ and uniformly distributed in the set {−1,0} when $x_{k}=X$. $W_{k}^{\Delta y}$ is identically distributed to $W_{k}^{\Delta x}$.

Given the current state of the system $s_{k}$ and the control $u_{k}$ the state of the system at the beginning of the (k+1)-th stage will be,

(2) $$s_{k+1}=\left[\begin{array}{c} x_{k+1}\\ y_{k+1}\\ m_{k+1} \end{array}\right]=\left[\begin{array}{c} x_{k}+W_{k}^{\Delta x}\\ y_{k}+W_{k}^{\Delta x}\\ m_{k}-u_{k}^{m}+W_{k}^{m} \end{array}\right].$$

We note in Equation (2) that in order to derive $m_{k+1}$ we subtract from $m_{k}$ all $u_{k}^{m}$ packets selected for transmission. This is equivalent to stating that all transmissions attempted within stage *k* are successful or considered successful by the mobile node. A mobile node will always consider a transmission successful, i.e., it will not attempt to retransmit a packet, when it does not require an acknowledgment of reception from the base station. This latter assumption regarding the configuration of the mobile node is justified by the fact that our focus is on IoT applications where energy efficiency is the major priority. To this end we consider the most energy efficient configuration for both the LP-WAN and LP-PAN protocols. This partially accounts to selecting the Aloha protocol for medium access control (MAC) and transmitting packets without requiring an acknowledgment of reception. The proposed configuration is supported by the 802.15.4 standard ([28], Section 4.5.4.2-3) for LP-PANs and is a typical configuration for LP-WANs [29].

With every state transition, as driven by control $u_{k}$, there is an associated cost. In the case of packet transmissions the associated cost is the amount of energy required to transmit $u^{m}$ packets over the selected interface. In the case of packet drops we assume a virtual energy cost per packet to prevent the mobile node from dropping packets freely. We denote with $E_{l}$ and $E_{w}$ the energy cost for the transmission of a single packet by use of the LP-PAN and LP-WAN interfaces, respectively, and with $E_{d}$ the virtual energy cost related to a packet drop. We assume that $E_{l}$ and $E_{w}$ are fixed for all cells and equal to the average energy consumption induced to the mobile node for the transmission of a packet for a given interface. This assumption is justified by the fact that $E_{l}$ and $E_{w}$ are uncontrollable system parameters within the context of our work. Equation (3) presents the state transition cost as described above,

(3) $$g\left(s_{k},u_{k}\right)=u_{m}\cdot \left[\left(1-u_{k}^{w}\right)E_{l}+\left(1-u_{k}^{p}\right)E_{w}+\left(1-u_{k}^{p}\right)\left(1-u_{k}^{w}\right)E_{d}\right].$$

We are interested in minimizing the total energy consumption accumulated over an horizon, i.e., an number of stages, expressed as follows,

(4) $$J_{\pi }\left(s_{0}\right)=\lim_{N\to \infty }\underset{\underset{k=0,1,\cdots }{W_{k}}}{E}\left\{\sum_{k=0}^{N-1}\gamma^{k}g\left(s_{k},u_{k}\right)|s_{0}\right\},$$

where $s_{0}$ is the initial state of the system, expectation is taken with respect to the joint probability mass function of the random variables comprising $W_{k}=\left(W_{k}^{m},W_{k}^{\Delta x},W_{k}^{\Delta y}\right)$ and γ is a discount factor, i.e., 0<γ<1, indicating that the importance of energy consumption decreases with time. Finally, π represents a policy, i.e., a sequence of functions $\pi =\{\mu_{0},\mu_{1},\cdots \}$, where each function $\mu_{k}$ maps states to controls for the *k*-th stage. For a policy π to belong to the set of all admissible policies Π, functions $\mu_{k}$ must satisfy the constraint that for stage *k* and state $s_{k}$ controls are selected exclusively from the set $U(s_{k})$.

In order to minimize Equation (4) we must apply an appropriate control $u_{k}$ at each stage *k* given state $s_{k}$. However, decisions cannot be viewed in isolation since one must balance the desire for low cost at the current stage with the avoidance of high future costs due possibly unfavorable state transitions. For example, the mobile node might avoid to transmit packets at the current stage and state, a decision that will always result in zero energy consumption, yet, due to its random walk, at the next stage it may find itself in a cell without network connectivity and a full backlog due to new packet arrivals. As a result of its last decision the mobile node will now have to drop packets, possibly at an excessive cost.

## 4. Transmission Policy

To deal with the dynamic program presented in Section 3 we capitalize on the computational methods provided by the framework of MDP The problem at hand constitutes a MDP since the state and decision spaces are finite, the distributions of the random system parameters are independent of each other, independent of their past values and invariant over time, i.e., the system is stationary and as a result state transitions are independent of past states and controls, given the current state $s_{k}$ and control $u_{k}$, and finally, the cost function is additive over time. The MDP can be described by the probability of transition between states,

(5) $$P\left\{s_{k+1}=j|s_{k}=i,\;u_{k}=u\right\}=p_{ij}\left(u\right)=P\left\{x_{k+1}|x_{k}\right\}\cdot P\left\{y_{k+1}|y_{k}\right\}\cdot P\left\{m_{k+1}|m_{k},u_{k}\right\}.$$

From this point on we will utilize the Markov Chain notation introduced in Equation (5), whereby $p_{ij}\left(u\right)$ is the probability that the system will make a transition to state *j* given that the system is in state *i* and decision *u* was made. For the MDP under consideration, given that 0<γ<1, there exists an optimal stationary policy π={μ,μ,⋯}, i.e., a policy that applies the same control function μ at all stages ([30] Section 2.3). What is more, the control function μ will be independent of the initial state of the system and deterministic [30], i.e., each time the system is in state *i*, μ(i) will make the same decision *u*. We will refer to a stationary policy π={μ,μ,⋯} as stationary policy μ. Our objective is to find a stationary policy $\mu^{*}$, from the set of all admissible stationary policies M⊆Π, that minimizes total cost in Equation (4), i.e.,

(6) $$\mu^{*}=\arg\min_{\mu \in M}J_{\mu }\left(i\right),\;\mathrm{for}\;\mathrm{all}\;i\in S.$$

Let $J^{*}$ be the total cost attained when the optimal policy $\mu^{*}$ is used, then, for the MDP at hand, $J^{*}$ satisfies the Bellman equation,

(7) $$\begin{array}{cc} J^{*}\left(i\right) & =\min_{u\in U(i)}\sum_{j=1}^{n}p_{ij}\left(u\right)\left[g\left(i,u,j\right)+\gamma J^{*}\left(j\right)\right],\;\mathrm{for}\;\mathrm{all}\;i\in S,\\ & =\min_{u\in U(i)}\left[g\left(i,u\right)+\gamma \sum_{j=1}^{n}p_{ij}\left(u\right)J^{*}\left(j\right)\right],\;\mathrm{for}\;\mathrm{all}\;i\in S, \end{array}$$

where *n* is the cardinality of the state space. Equation (7) actually describes a system of *n* non-linear equations, the right hand side of which is a contraction with a unique fixed point located at $J^{*}\left(i\right)$. Due to the contraction property one can derive both $J^{*}$ and $\mu^{*}$ via iterative methods. In this work we utilize the Optimistic Policy Iteration (OPI) algorithm [30,31,32] to approximate the optimal policy $\mu^{*}$ and the optimal horizon cost $J^{*}$.

The OPI algorithm depends on the Approximate Policy Evaluation (APE) algorithm thus we begin with the description of the latter algorithm and then proceed with the description of the OPI algorithm itself. Given a policy μ we can approximate the horizon cost $J_{\mu }$ by use of the APE algorithm presented in recursive form in Algorithm 1.

Formally, Algorithm 1 converges to $J_{\mu }$ only after an number of recursive calls, i.e., when $r_{\max}\to \infty$. In practice, however, a finite value for $r_{\max}$ must be selected and heuristically chosen values for $r_{\max}$ lead to an accurate calculation of $J_{\mu }$ as indicated by analysis and computational experience [30]. Another popular variation of Algorithm 1 stops the sequence of recursive calls when $\max_{i\in S}\left|J_{\mu }^{\prime }\left(i\right)-J_{\mu }\left(i\right)\right|$ becomes smaller than a predefined threshold [31].

**Algorithm 1** Approximate Policy Evaluation
**Require:**
μ∈M
 1:Initialize $J_{\mu }\left(\cdot \right)$ arbitrarily 2:
r←0
 3:**Function** policy_evaluation(μ, $J_{\mu }$, *r*) 4:
**if** $r<r_{\max}$ **then**
 5:  $J_{\mu }^{\prime }\left(i\right)\leftarrow g\left(i,\mu \left(i\right)\right)+\gamma \sum_{j=0}^{n}p_{ij}\left(\mu \left(i\right)\right)J_{\mu }\left(j\right)$, for all i∈S 6:  policy_evaluation(μ, $J_{\mu }^{\prime }$, r+1) 7:
**else**
 8:  {*r* is equal to $r_{\max}$} 9:  Return $J_{\mu }$10:
**end if**



The OPI algorithm is presented in Algorithm 2. The major operation of the OPI algorithm, besides calling APE, is presented in Line 3 and is typically called the *policy improvement* step because its execution results in an a policy that has a smaller horizon cost. The intuition behind the policy improvement step is that we can improve the current policy by finding a control *u*, available at the current state *i*, that reduces the immediate transition cost g(i,u) as well as the expected future cost, which, however involves $J_{q}$, i.e., it has been estimated with the previous, non-improved policy. According to the Bellman’s optimality principle [32,33], unless policy $\mu_{q}$ is the optimal policy, the policy improvement step will result in a better control for at least one state. Formally, Algorithm 2 will converge to $\mu^{*}$ as $q_{\max}$ goes to infinity, even if the Policy Evaluation algorithm is executed for a finite value of $q_{\max}$. However, similar to the APE algorithm, analysis and computational experience [30] suggest that heuristically selected finite values of $q_{\max}$ approximate $\mu^{*}$ and its corresponding optimal cost $J^{*}$ with adequate accuracy.

**Algorithm 2** Optimistic Policy Iteration
1:Initialize $J_{1}$ arbitrarily and r←0.2:**for**q=1 to $q_{\max}$ **do**3:  $\mu_{q}\left(i\right)=\arg\min_{u\in U(i)}\left[g\left(i,u\right)+\gamma \sum_{j=0}^{n}p_{ij}\left(u\right)J_{q}\left(j\right)\right]$, ∀i∈S4:  $J_{\mu_{q}}$ = policy_evaluation($\mu_{q}$, $J_{q}$, *r*)5:  $J_{q+1}=J_{\mu_{q}}$6:
**end for**
7:Return $\mu_{q}$ and $J_{\mu_{q}}$


A significant drawback of Algorithm 2 is that its computational complexity is polynomial in $\left|S\right|\times \left|U\right|\times q_{\max}$, where |·| represents the cardinality of the enclosed set. The number of states |S| for the problem at hand is X×Y×M, i.e., the number of states increases exponentially with the number of cells and the size of the backlog. The resulting exponential growth in computational complexity is typically referred to as the curse of dimensionality and plagues all dynamic programming problems. Thus, to tackle larger instances of the problem at hand we have to resort to computationally efficient albeit suboptimal algorithms. To this end we utilize the Rollout Algorithm [30,34], a limited lookahead algorithm, that is based on the OPI algorithm presented above. In the Rollout algorithm, instead of initializing $J_{0}$ arbitrarily we begin with a heuristic policy $\mu_{h}\in M$ and derive its cost $J_{h}$. A widely used heuristic policy is the myopic policy that selects control *u* at state *i* so that g(i,u) is minimized and completely disregards the expected cost due to future state transitions as expressed by $\sum_{j=0}^{n}p_{ij}\left(u\right)J_{\mu }\left(j\right)$, i.e.,

(8) $$\mu_{h}\left(i\right)=\arg\min_{u\in U(i)}g\left(i,u\right),\;\mathrm{for}\;\mathrm{all}\;i\in S.$$

Following the estimation of $J_{h}$ one or more policy improvement steps are applied to produce an improved policy. The Rollout Algorithm is presented in Algorithm 3.

**Algorithm 3** Rollout Algorithm
 1:Define heuristic policy $\mu_{h}\in M$; r←0 2:$J_{h}$ = policy_evaluation($\mu_{h}$, $J_{h}$, *r*) 3:$J_{1}$ = $J_{h}$ 4:**for**q=1 to $q_{\max}$ **do** 5:  { $q_{\max}$ is typically small, e.g., 1 or 2} 6:  $\mu_{q}\left(i\right)=\arg\min_{u\in U(i)}\left[g\left(i,u\right)+\gamma \sum_{j=0}^{n}p_{ij}\left(u\right)J_{q}\left(j\right)\right]$, ∀i∈S 7:  $J_{\mu_{q}}$ = policy_evaluation($\mu_{q}$, $J_{q}$) 8:  $J_{q+1}=J_{\mu_{q}}$ 9:
**end for**
10:Return $\mu_{q}$ and $J_{\mu_{q}}$


## 5. Results

In this section we evaluate numerically the energy efficiency of the packet transmission policies produced by the OPI and Rollout algorithms. To facilitate insight into the energy efficiency of the aforementioned policies we introduce another heuristic policy, which is often used in contemporary DTNs, whereby the mobile node will transmit every packet in its backlog whenever a network connection is available. In case more than one network connections are available in a cell the mobile node will use the most energy efficient one and, in case no network connection is available in a cell, it will drop packets in order to be able to accommodate $W_{m}^{\max}$ incoming packets. We term this policy the Empty-Backlog policy.

We begin with the illustrative example presented in Figure 1 that involves a surveillance area of 20×20 cells, i.e., X=20 and Y=20, which includes five LP-WAN base stations and nine LP-PAN base stations. LP-PAN and LP-WAN base stations have a communication range that extends to an euclidean distance of one and five cells respectively. The capacity of the mobile node’s backlog is nine packets and the maximum number of packet arrivals within a stage is uniformly distributed between zero and three packets, i.e., $W_{m}^{\max}=3$. We assume that the transmission of a single packet through a LP-PAN and a LP-WAN connection costs one and two energy units respectively, while packet drops account for ten energy units. Finally, we set the discount factor to 0.9.

**Figure 2:**
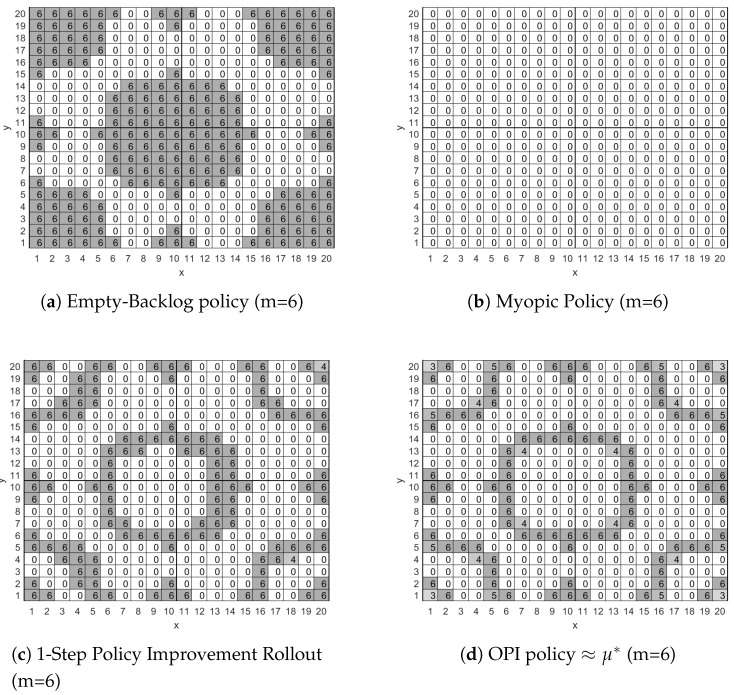
Packet transmission policies for the scenario of Figure 1 for the set of states with m=6, i.e., with six packets stored in the backlog. The policy produced by the Rollout algorithm with two policy improvement steps is identical to the policy produced by the optimistic policy iteration (OPI) algorithm.

Figure 2 presents, for four different policies, the number of packets $u^{m}$ that the mobile node will have to transmit in each cell whenever there are six packets in its backlog. To facilitate presentation we do not include in Figure 2 the values of $u^{w}$ and $u_{l}$ since for all four policies the mobile node will use the most energy efficient network connection available in each cell.

More specifically, in Figure 2a we present the Empty-Backlog Policy according to which the mobile node will transmit all six packets stored in the backlog whenever the node enters a cell that has network connectivity, i.e., a value of 1, 2 or 3 in Figure 1. The policy does not involve any packet drops for cells with no network connectivity, i.e., cells with a value of 0 in Figure 1, because the backlog’s capacity is nine packets and only six packets are stored in it, thus there is enough space for the worst case scenario of three new packet arrivals ($W_{m}^{\max}=3$) at the current stage.

In Figure 2b we present the myopic policy according to which the mobile node will not transmit in any cell of the surveillance area, since, myopically, this is the most energy efficient thing to do. We note again here that only six packets are stored in the backlog and thus there is enough space to accommodate the worst case scenario of three new packet arrivals ($W_{m}^{\max}=3$), thus the mobile node is not forced to transmit or drop packets.

In Figure 2c we present the policy derived by the Rollout algorithm with a single policy improvement step. As seen in Figure 2c, the mobile node will transmit all packets in its backlog whenever it enters a cell that lies on the edge of an area with network connectivity. This is due to the Rollout algorithm identifying that the mobile node will probably make a transition to a region that offers no network connectivity where costly packet drops are the only way to empty the backlog in order to collect new packets. On the other hand, while the mobile node is within an area with network coverage it will transmit packets preferably when the most energy efficient interface is available, e.g., when in cell (1,1), and will avoid transmitting packets otherwise, e.g., when in cell (3,1).

Finally, in Figure 2d we present the approximately optimal policy produced by the OPI algorithm when executed with $r_{\max}=50$ and $q_{\max}=50$. The OPI algorithm’s policy is an improved version of the policy presented in Figure 2c. Their main difference is a small reduction in the number of packets to be transmitted when in cells that lead with high probability to a neighboring cell that offers LP-PAN network connectivity, e.g., cells (1,1), (1,20) and (20,1), and cells that lead with low probability to an area with no network connectivity, e.g., cell (5,1). A major result of this work is that the policy derived by the Rollout algorithm with two policy improvement steps is identical to the policy derived with the OPI algorithm. The importance of this result is that we may attain an approximately optimal policy by utilizing a computationally efficient suboptimal algorithm.

Finally, although we do not present the relevant figures here due to lack of space, we note that when the number of packets in the backlog is less than six all four policies will retain their form, as presented in Figure 2, although the number of transmitted or dropped packets in each cell will decrease. Obviously, when the backlog is empty no packet will be transmitted at any cell for all policies. On the other hand, when the mobile node has more than six packets in its backlog then it is obliged by the queue management policy to transmit or drop packets in each cell so that there is room for $W_{m}^{\max}$ packets in the backlog. Thus, for the case of seven, eight and nine packets in the backlog all four policies will retain their previous form, as presented in Figure 2, although the number of transmitted or dropped packets in each cell will increase respectively by one, two and three.

**Figure 3:**
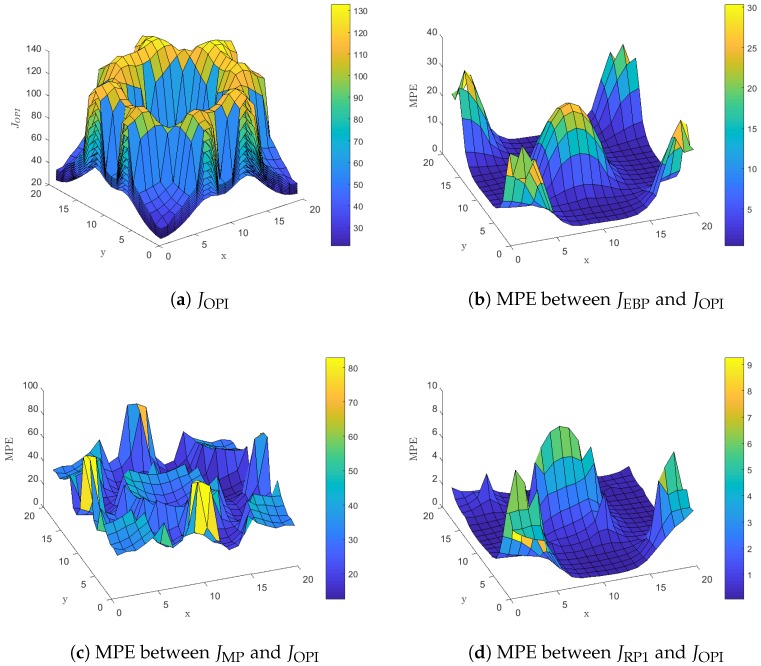
Mean Percentage Error (MPE) in energy consumption for each suboptimal policy compared to the horizon discounted cost for the Optimistic Policy Iteration (OPI) policy $J_{\mathrm{OPI}}$.

Figure 3 presents results related to the expected energy cost over an horizon for all policies presented in Figure 2. More specifically, Figure 3a presents $J_{\mathrm{OPI}}$ the expected discounted cost over an horizon for each state of the mobile node when the approximately optimal OPI policy is used. The figure includes nine surface plots, one for each state of the backlog *m*, that cover the whole state space *S*. The surface plot that lies on top of all others corresponds to the case where m=9, i.e., the expected energy cost is maximized when the backlog is full. As the number of packets in the backlog decreases we get lower values for the expected energy cost in all cells and the minimum is attained when the backlog is empty. Furthermore, Figure 3a indicates that the expected energy cost is small for cells that lie in the vicinity or within the service area of a LP-PAN, increases for cells in the vicinity or within the service area of a LP-WAN and becomes maximum for cells that lie outside the communication range of any base station.

Figure 3b presents the mean percentage error (MPE) [35] between $J_{\mathrm{EBP}}$, the horizon discounted cost when the Empty-Backlog Policy is used, and $J_{\mathrm{OPI}}$ calculated as

(9) $$\mathrm{MPE}\left(x,y\right)=\frac{1}{M}\sum_{m=0}^{M}\frac{J_{\mathrm{EBP}}\left(x,y,m\right)-J_{\mathrm{OPI}}\left(x,y,m\right)}{J_{\mathrm{OPI}}\left(x,y,m\right)}\cdot 100\;\left(\%\right).$$

Equation (9) is well defined because $J_{\mathrm{OPI}}$ is a positive real number. Furthermore, the quantity in the MPE sum represents the percentage *increment* in cost due to using a suboptimal policy since $J_{\mathrm{EBP}}\geq J_{\mathrm{OPI}}$ for all states. Finally, in Equation (9) we average over all possible backlog values in order to avoid presenting a different surface plot for each state of the backlog as we did in Figure 3a. Now, Figure 3b indicates that the Empty-Backlog Policy does not utilize the heterogeneity of the available networks within the surveillance area. Instead, the mobile node blindly transmits all packets in the backlog whenever it enters a cell with LP-WAN coverage disregarding the fact that neighboring cells may offer a more energy efficient network service such as LP-PAN. This results in increments of expected energy cost up to 30% for cells that lie in the vicinity of LP-PAN base stations.

Similarly, Figure 3c presents the MPE between the $J_{\mathrm{MP}}$, the horizon discounted cost for the Myopic Policy, and $J_{\mathrm{OPI}}$. The myopic policy performs poorly in general, with increments in energy cost as high as 80%. This is mainly due to its short term energy conserving policy that focuses entirely on saving energy at the current stage and completely disregards the potential transitions to cells with only LP-WAN connectivity or no connectivity at all at subsequent stages. Actually, transmissions occur only when the policy is forced to do them, i.e., when the backlog has more than six packets, and this will often result in packet drops at an excessive cost.

Figure 3d presents the MPE between the $J_{\mathrm{RP}1}$, the horizon discounted cost for the policy derived by the Rollout Algorithm with one policy improvement step, and $J_{\mathrm{OPI}}$. As described previously in this section the Rollout Algorithm with one policy improvement step is only slightly different from the OPI policy. This is reflected in the low values for ${\mathrm{MPE}}_{\mathrm{RP}1,\;\mathrm{OPI}}$, presented in Figure 3d, compared to the previous two policies. For ${\mathrm{MPE}}_{\mathrm{RP}1,\;\mathrm{OPI}}$, increments in energy cost are mainly due to transmitting a larger number of packets, compared to the approximately optimal policy, in cells such as (1,1), (1,20), (20,1) and (20,20). This increment in the number of transmitted packets does not actually increase energy consumption, since in all these cells an LP-PAN is available and packet transmission are conducted with the minimum energy expense. However, we observe an increment in energy cost because the Rollout Algorithm with one policy improvement step disregards the effect of the discounting factor γ on energy cost. The OPI policy will favor delayed packet transmissions, whenever this does not lead with high probability to energy inefficient packet transmissions or packet drops in future stages, because future energy costs are *discounted*.

**Figure 4:**
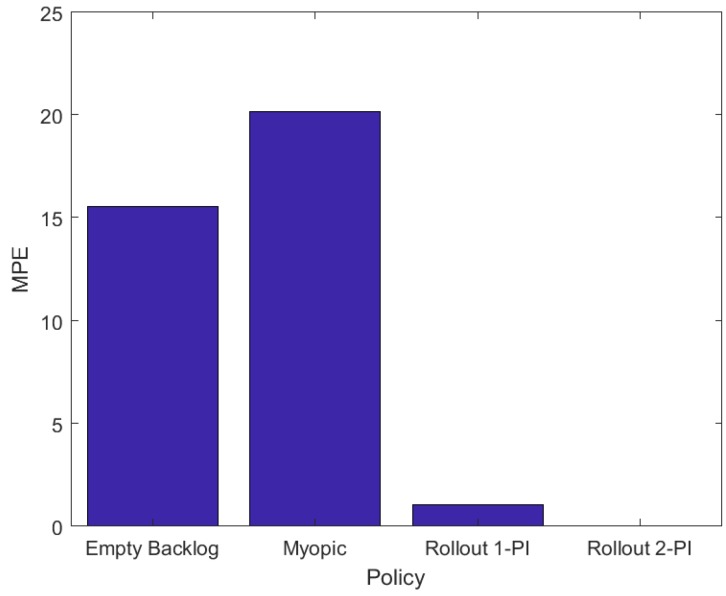
Mean percentage error increment in energy consumption for the Empty Backlog, Myopic and Rollout, with one and two policy improvement steps, policies, compared to the OPI policy.

To assess the energy efficiency of all the aforementioned policies in a variety of base station topologies we conducted two thousand numerical experiments for a surveillance area of 20×20 cells that includes five LP-WAN base stations and five LP-PAN base stations. In each numerical experiment the base stations were placed randomly within the surveillance area. Other than the number and the placement of the base stations the configuration is identical to that of the illustrative example presented above. Figure 4 presents MPE in energy consumption for the four policies, calculated as follows,

(10) $${\mathrm{MPE}}_{P}=\frac{1}{|S|\cdot L}\left(\sum_{l\in L,s\in S}\frac{J_{P}^{l}\left(s\right)-J_{\mathrm{OPI}}^{l}\left(s\right)}{J_{\mathrm{OPI}}^{l}\left(s\right)}\right)\cdot 100\;\left(\%\right),$$

where *L* is the number of numerical experiments, |S| is the cardinality of the state space and $J_{P}$ is one of $J_{\mathrm{BEP}}$, $J_{\mathrm{MP}}$, $J_{\mathrm{RP}1}$ and $J_{\mathrm{RP}2}$. In Equation (10) the mean value for the percentage error is calculated over all states of the system and, subsequently, over all experiments. Figure 4 depicts that the Myopic and Empty-Backlog policies result in a significant increment in energy consumption compared to the OPI policy. On the other hand, the Rollout algorithm with one policy improvement step significantly reduces the energy cost compared the myopic policy, its base heuristic policy, and achieves an approximately optimal energy cost with just two policy improvement steps. The results of Figure 4 exhibit the significance of the network availability map and localization information in devising energy efficient packet transmission policies for mobile nodes in DTNs. In comparison, policies that rely only on local network availability information, although easier to implement, lead to a significant increase in energy cost and packet drops.

## 6. Conclusions and Future Plans

In a large number of IoT applications end nodes suffer intermittent connectivity due to mobility and sparse network coverage. Heterogeneous networks and DTNs have emerged as potential solutions to this problem. In this work we formulated the problem of finding energy efficient packet transmission policies, under the constraint of finite data storage, as a dynamic programming problem and utilized the framework of Markov Decision Processes to derive approximately optimal and suboptimal data transmission policies. Finally, we evaluated numerically the derived policies and showed that significant gains can be achieved by utilizing the derived approximately optimal and suboptimal policies.

Future research activities include a revision of the proposed scheme to transform it to become more dynamic and adaptive, able to consider also the temporal variation of the network availability map. Additionally, future plans include the design of a variation of the proposed scheme to work with cognitive radio inspired IoT nodes (extending our previous works described in [36,37] that have the ability to sense the spectrum and extract the network availability map without having to know the map beforehand. Finally, we are also working towards exploiting deep reinforcement learning to solve the problem at hand aiming to learn what is the optimal transmission policy for each scenario.
